# A critical review of conventional and emerging technologies for the detection of contaminants, allergens and adulterants in plant-based milk alternatives

**DOI:** 10.1016/j.crfs.2025.101067

**Published:** 2025-05-08

**Authors:** Zahra Karimi, Katrina Campbell, Zoltan Kevei, Andrea Patriarca, Anastasios Koidis, Maria Anastasiadi

**Affiliations:** a Faculty of Engineering and Applied Sciences, Cranfield University, College Road, Cranfield, MK43 0AL, United Kingdom; b Institute for Global Food Security, School of Biological Sciences, Queen's University of Belfast, 19 Chorine Gardens, Belfast, BT9 5BN, United Kingdom

**Keywords:** Plant-based milk alternatives, Contaminants, Allergens, Adulterants, Portable methods, Limits of detections, DNA based methods

## Abstract

The increasing popularity of plant-based milk alternatives (PBMAs) necessitates effective safety and authentication measures to ensure food product integrity and maintain consumer trust. This review aims to offer a comprehensive overview of potential contaminants, allergens, and adulterants in PBMAs, and the analytical methodologies employed for their detection and quantitation. It details the advantages and limitations of widely employed testing techniques, such as chromatography, spectroscopy, immunoassays and PCR. In addition, it explores recent advancements in portable detection methods based on novel technologies such as CRISPR and biosensor systems that offer new opportunities for rapid and precise analysis. Despite these technological innovations, important challenges remain, particularly in optimizing sample preparation protocols and improving DNA-based methods efficiency. The integration of multiple detection strategies and the development of rapid, cost-effective analytical tools are critical steps towards enhancing both industry compliance and consumer confidence. Furthermore, green analytical methods — such as solvent-free extraction, AI-driven spectroscopy, and sustainable sample preparation techniques — pave the way toward eco-friendly and more efficient PBMA safety testing.

## Highlights

• PBMAs risk contamination from biological and chemical sources like microbes and toxins.
• Various advanced and emerging methods detect PBMAs contamination and adulteration.
• Limit of detection and quantification of current methods for PBMAs safety.
• Research gaps exist in detecting viral, and processing-related contaminants.
• Innovative tech is key to closing research gaps and ensuring PBMA safety and quality.

## Graphical abstract

**Figure:**
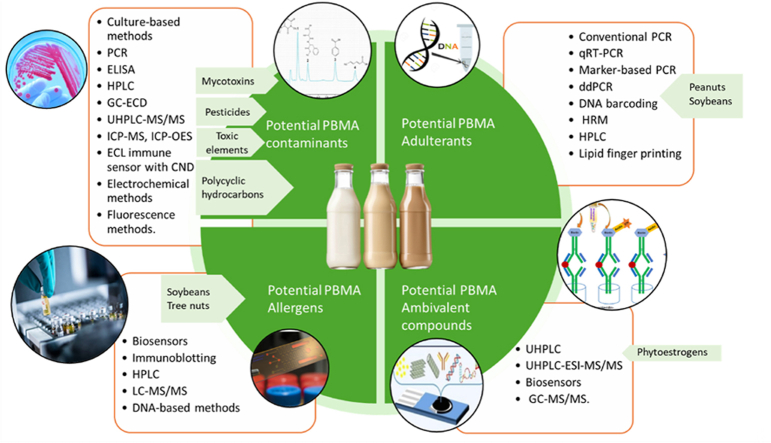

## 1 Introduction

Plant-based milk alternatives (PBMAs) are obtained from water-based plant extracts by separating the liquid part from the solid particles through i) disruption of tree nuts, seeds or legumes to make aqueous suspension of oil bodies; and ii) creation of oil-in-water emulsions by thehomogenisation of oil, water and emulsifier. In recent years, these products have attracted interest from the food industry due to high consumer demand for alternatives to animal milk (Daryani et al., 2024; Sethi et al., 2016). Since PBMAs are designed to mimic the physicochemical, nutritional, and sensory attributes of bovine milk, consumption of this product as an alternative to conventional animal milk has grown dramatically over the past few years. In this context, factors such as the rapid increase in vegan diet preferences, bovine milk allergies, lactose intolerance, calorie concerns, environmental awareness and sustainability have created a rapidly increasing demand for PBMAs. The value of PBMAs sales is currently estimated at USD 12.1 billion and is predicted to reach USD 29.5 billion by 2031 with a growth rate of 10.18 % from 2020 to 2024 (Chaiwanon et al., 2000; Daryani et al., 2024; Romulo, 2022).

Currently, commercially available PBMA products are classified as legume-based, cereal-based, nut-based, seed-based, and pseudocereal-based milks. The process of producing PBMAs involves multiple steps: soaking, milling and grinding for the reduction of the size of raw material, then filtration, formulation, fortification, homogenization and finally ultra heat treatment (UHT) sterilization. This process is largely similar for all types of PBMA products with some minor differences (Reyes-Jurado et al., 2023; Romulo, 2022).

**Fig. 1:**
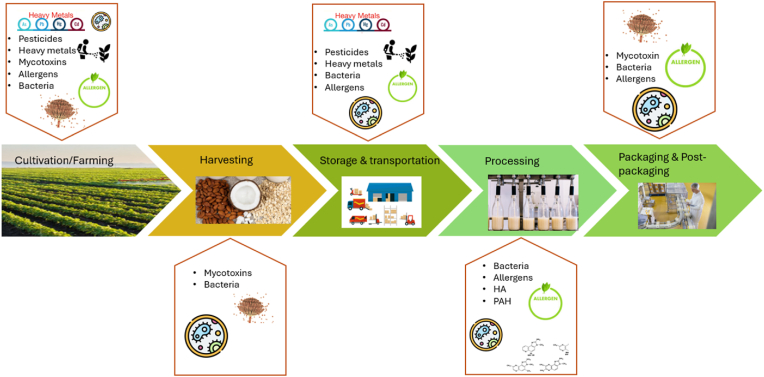
A visual representation of the production stages of PBMAs and the potential points of contamination and allergen introduction. This diagram illustrates the five major stages of PBMA production—Cultivation/Farming, Harvesting, Storage & Transportation, Processing, and Packaging & Post-Packaging—with associated risks of chemical, biological, and natural contaminants as well as allergenic compounds.

Previous research studies on PBMAs have mostly focused on improving physicochemical characteristics, such as texture, flavour, and appearance of PBMA analogs, as well as nutritional aspects (McClements et al., 2019). Furthermore, previous research has considered the rapid increase in PBMA consumption driven by consumer demand for a healthy and sustainable diet and the safety concerns associated with this trend. All food products -PBMAs are not the exception-may carry numerous and varied chemical and biological contaminants which may imply serious harm to human health. Moreover, intentional adulteration or cross-contamination with plant and possibly animal ingredients which are not declared on the ingredient list of PBMAs may also result in allergic reactions posing a risk to consumers' health. Food fraud is a particular concern in the food supply chain and fraudulent practices of any kind are illegal and undermine consumers' trust. Thus, safe consumption of PBMAs relies on preventing contamination and adulteration at any stage of the supply chain, from crop development, production of raw materials and processing stages to the final product. Identifying potential contamination sources and developing sensitive and accurate methods to detect adulteration in PBMAs is therefore crucial for ensuring the integrity and safety of PBMAs products available in the market (Fig. 1.). This information can be useful for preventing food fraud, ensuring the development of commercial PBMAs on safety and quality, protecting consumer's health, and improving the establishment of relevant standards by food safety authorities.

**Fig. 2:**
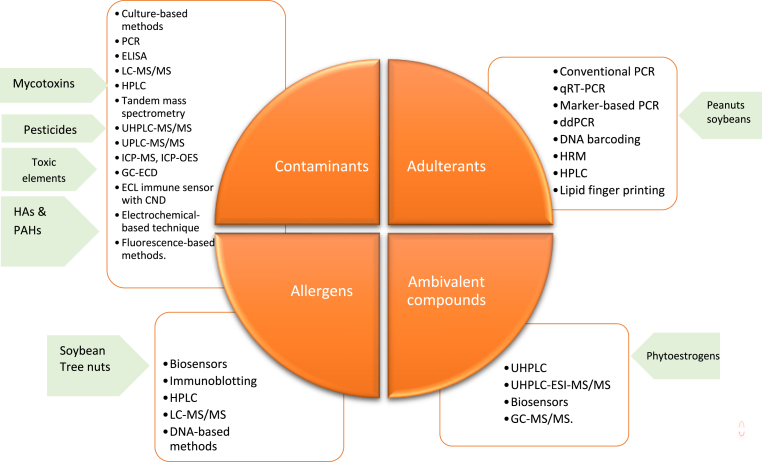
Overview of the main/investigated PBMAs adulterants, contaminations, allergens and ambivalent compounds and detection methods. HA – heterocyclic aromatic amines PAH – polycyclic aromatic hydocarbons

The main body of review studies on plant-based food safety challenges have focused on the occurrence of toxins, such as natural toxins in plant-based meat and milk alternatives, the potential food safety hazards associated with plant-based alternative proteins and the source, occurrence and detection methods for plant-based foods (Augustin Mihalache et al., 2022, Banach et al., 2022, Lin et al., 2023). However, to the best of our knowledge, there is no comprehensive review to date detailing potential PBMA contamination, adulteration and allergen risks and the associated chemical and biological detection methods. In order to ensure that PBMAs are safe and suitable for consumption, it is imperative to develop an in-depth understanding of the risks related to biological and chemical contamination and/or adulteration of these products. The aim of this review is to provide information on potential contamination sources and adulteration practices in PBMAs and review established and emerging analytical and molecular-based detection methods (Fig. 2.). We envisage that the present study could be a useful reference for improving the standards, quality and safety of PBMA products and act as an essential guidance for regional and national food safety authorities.

## 2 PBMA contaminant types

PBMA contamination sources comprise of biological (e.g. bacterial, mycotoxins, fungal, viral and antibacterial), and chemical contaminants (e.g., pesticides and toxic elements) as well as ambivalent compounds with both positive and negative health effects. In addition to these micro-contaminants, macro-contaminants or foreign organic material, such as insect fragments, and mammalian hair, have also been investigated using microscopy methods. This approach can be challenging due to the similarity between histological elements of different plants in the same sample. It is not a simple method and has limitations in identifying mixtures or highly diluted ingredients (Fioravanti et al., 2024). This review primarily focuses on microcontaminants, and the detection methods for each type are detailed in the following chapters.

### 2.1 Microbial contaminants

Microorganisms, including bacteria, fungi and viruses, along with the antibacterial compounds they can produce are the primary agents responsible for both spoilage and pathogenic effects in food products.

PBMAs are heat-treated products that typically undergo commercial sterilization using UHT methods (138–145 °C for a short time 1–10 s). This process is mainly intended to eradicate spores of spoilage-causing and pathogenic microorganisms such as bacteria, ensuring microbiological safety and extending the product's shelf life. Despite the severity of the thermal process it has been shown to be insufficient in eradicating spores of thermophilic spore-forming microorganisms, which include highly heat-resistant endospores (Sethi et al., 2016).

Fungal contamination of PBMAs can occur at various stages in the supply chain, from the field where raw materials are sourced, to processing facilities, and even in consumers' homes. This is because PBMAs provide an environment with high moisture and suitable temperature conditions which can promote fungal growth. Most yeast and mould species, with a few exceptions, are not heat-resistant and should be eliminated during pasteurization but their thermal resistant spores may persist. Also, fungal contamination during production can occur after the heat treatment of the product. Mould spoilage is frequently caused by airborne fungi, as fungal spores can easily spread through the air in a processing environment (Garnier et al., 2017; Kure et al., 2004).

Although the risk of antibiotic residues in plant material is significantly lower compared to animal products, traces have been also reported in plant-based foods. This is because plants can absorb antibiotics from the environment resulting in antibiotic accumulation within the plants (Bacanlı, 2024; Piña et al., 2020). The most common crops that can accumulate antibiotics are cereals like wheat, rice, and oats, which may also transmit these residues to the final products, such as PBMAs (Ghimpețeanu et al., 2022). However, research on detecting viral and fungal contamination (such as spoilage yeasts and moulds) in PBMAs as well as antibiotic and drug residues is still lacking. The following section describes some of the most recent studies on PBMA microbial contamination, including pathogenic and spoilage bacteria, and the various methods used for their detection.

Pathogenic bacteria represent a serious health hazard, as they can lead to illness even in minimal concentrations. These bacteria, including genera like *Salmonella*, *E. coli*, and *Listeria*, can contaminate food products leading to severe illness. In addition to the dangers posed by pathogens, food spoilage bacteria are also of significant concern. These can degrade the quality and safety of food, leading to off-flavours, off-odours and textures that make the product unfit for consumption. Spoilage bacteria can also create an environment that allows other pathogens to thrive, further increasing the risk of foodborne illnesses.

Together pathogenic and spoilage bacteria present substantial challenges to food safety, emphasizing the need for vigilant monitoring. Several studies have explored the potential microbial contamination of PBMAs. Giugliano et al. (2023) investigated pathogen bacterial contamination in soy, oat and rice milk using culture-based methods and showed no contamination in any of the samples, indicating that thermal processes are sufficient for microbiological safety (Giugliano et al., 2023). Another study investigated the growth rate of food spoilage and pathogen microbes using culture-based methods in coconut, almond and cashew milk (Bartula et al., 2023). They compared the colony forming counts (CFU) of *Listeria* and *Salmonella* (food-borne pathogens) and *Paenibacillus* and *Bacillus* (spoilage spore-forming microbes) after inoculating commercial PBMAs and bovine milk stored at different temperatures (4, 8, 20 °C). It was found that *Listeria* (at 8 °C and 20 °C), *Salmonella* and *Paenibacillus* (at 20 °C) grew better in PBMAs compared to bovine milk, while *Bacillus subtilis* proliferated equally on both products. Based on these findings it was concluded that there is significantly higher risk of *listeriosis* and *salmonellosis* in PBMAs compared to bovine milk if the thermal treatment fails or in the occurrence of post-processing cross-contamination.

A recent study by Qian et al. (2022) introduced a novel portable detection platform based on CRISPR/Cas12a method to detect *Staphylococcus aureus (S. aureus)* in commercial soy milk and other plant-based foods. Traditional methods for detecting *S. aureus*, such as culture-based techniques and PCR, are time-consuming, labour-intensive, and require specialised tools and expertise. The proposed CRISPR/Cas12a detection method integrates nucleic acid isothermal amplification using lateral flow strips for signal readout of the *nuc* target gene of *S. aureus* and a conserved 16 S rDNA fragment of the *Staphylococcus* genus. Crucially this CRISPR detection platform offers a combination of ultra-sensitivity, specificity, accuracy, portability, user-friendliness and time efficiency, meeting the practical requirements for point-of-care testing of *S. aureus* and other foodborne pathogens in future applications (Qian et al., 2022).

Spoilage bacteria detection has been targeted by Misiou et al. (2023), who performed a quantitative microbial spoilage risk assessment of PBMAs by the thermophilic spore-forming bacteria named *Geobacillus stearothermophilus* in Greece and Poland. Since UHT is primarily applied to remove spores of *Clostridium botulinum* from milk samples, the authors hypothesised that it may be insufficient for removing spores of thermophilic spore-forming bacteria. The risk of spoilage was assessed under the assumption that *G. stearothermophilus* reached its maximum concentration at the time of consumption and was measured based on current climate and future climate change scenarios. A model for the quantitative microbial spoilage risk assessment was created based on raw material contamination, heat inactivation of spores through UHT treatment, partitioning in the packs and growth of spores through distribution and storage of PBMAs. Their results showed that, under current climate conditions the risk of spoilage was negligible in Poland while it was considerable in Greece. However, under climate change scenarios, the risk of spoilage increased by up to 3-fold in Greece and showed significant increase for Poland (Misiou et al., 2023).

### 2.2 Mycotoxin detection

Despite no studies having been published to date on PBMA fungal contamination, there is a considerable amount of research available on mycotoxin contamination of PBMAs. Mycotoxins, a well-known group of toxic fungal secondary metabolites, can be transferred from raw plant sources to final plant-based products. These compounds are produced by certain types of mould that infect crop grains, nuts, and fruits, and can resist various food processing methods. Consequently, the presence of mycotoxins in raw materials poses a significant risk to the safety of PBMAs or other plant-derived food products; for example, aflatoxins generate significant health risks due to their carcinogenic effects (Santos et al., 2017). Understanding effective detection strategies for mycotoxin contamination is crucial for ensuring the safety and quality of plant-based foods. The following section reviews both analytical and immunological methods for mycotoxin detection.

#### 2.2.1 Analytical methods for qualitative and quantitative assessment of mycotoxins in PBMAs

Liquid chromatography (LC) coupled with mass spectrometry (MS) or a fluorescence detector (FLD) is the routine method for detecting and quantifying mycotoxins in food products. HPLC-FLD was used to develop a sensitive method for the simultaneous detection of five mycotoxins in soy milk (Rezaeefar et al., 2022) with the corresponding limit of detection (LOD) and limit of quantification (LOQ) reported in Table 1. Aflatoxin B1 was detected in all PBMA samples, with one sample also being contaminated by ochratoxin A (Rezaeefar et al., 2022). Another method assessed the presence of three mycotoxins (citrinin, zearalenone, and ochratoxin A) in oat and rice milk using in-line SPE-HPLC coupled with a fluorescence detector (Kholová et al., 2023). The authors compared twelve types of fibrous sorbents for inline extraction and chromatographic determination of the above mycotoxins. The validation parameters for this method, including LOD and LOQ are presented in Table 1. Zearalenone contamination detected in both oat and rice milk ranged from 9.5 to 14.6 μg/L, remaining within the tolerable limits defined by the European Union (20 μg/L, limits for processed cereal-based foods intended for children's consumption and red fermented rice food supplements) (Kholová et al., 2023; European Commission).

**Table 1:** A summary of mycotoxin determination methods for PBMAs.

Method	Sample preparation technique	Target mycotoxins	LOD (μg/L)	LOQ (μg/L)	Recovery (%)	RSD (%)	References
HPLC-FLD	carbon-based dispersive micro SPE combined with dispersive liquid–liquid microextraction	AFB1, B2, G1, G2, OTA,	0.13–0.83	0.43–2.7	70–87	<6.2	Rezaeefar et al. (2022)
HPLC	SPE	citrinin, zearalenone, OTA	0.09–3	0.3–10	72.1–106.1	<6.2	Kholová et al. (2023)
UHPLC-MS/MS	SALLE	enniatins, beauvericin	0.3	0.5–0.8	84–97	<8	Arroyo-Manzanares et al. (2019)
		fumonisin B1, B2, fusarenon-X HT-2, T-2, deoxynivalenol, zearalenone	3.2–57.7	NR^a^	>80	<12	Stanciu et al. (2017)
LC-MS/MS	QuEChERS	fumonisins B1 and B2, AFB1, B2, G1, and G2, OTA, zearalenone, oitreoviridin	0.007–2.428	0.02–8.09	72–107	4.08–16.87	Pinto et al. (2021)
ELISA	NR	AFB1, STC, OTA, DON, T-2/HT-2	0.08–16	0.008–0.95	≥90	NR	Rehagel et al. (2022a).
Indirect competitive MOFLISA	NR	AFB1	0.01–20	NR	86.41–99.74	2.38–9.04	Xu et al. (2021)
Immunochromatographic strip assays	NR	aflatoxins B1, B2, G1, G2, and M1	0.5	NR	NR	NR	Santos et al. (2017)

a NR: Not reported.

Arroyo-Manzanares et al. (2019) performed a study for the detection of emerging mycotoxins enniatins and beauvericins in commercial rice, oat and soy milk using UHPLC-MS/MS. The performance parameters are reported in Table 1. The results showed that 25 % of samples were contaminated with these types of mycotoxins with oat milk being more susceptible compared to rice and soy milk, with the latter showing no detectable contamination. The range of contamination was 0.4–26 μg/L with Enniatin B and B1 being present at the highest concentrations (Arroyo-Manzanares et al., 2019). In another study, UHPLC-MS/MS was also applied to determine mycotoxins of the *Fusarium* genus in oat, soybean, rice and “birdseed” PBMAs (Stanciu et al., 2017). The sensitivity, specificity and precision of this method for each matrix are shown in Table 1. Deoxynivalenol was detected in three commercial oat milks while other samples showed no detectable contamination with this group of mycotoxins. The presence of mycotoxins in oat milk was attributed to contamination of the raw materials (Stanciu et al., 2017).

In a study by Pinto et al. (2021), the detection of nine mycotoxins in commercial PBMAs was performed using LC-MS/MS. This method was coupled with QuEChERS as a cleanup technique to increase the efficiency of the mycotoxin extraction and reduce any background noise caused by matrix effects. The method was validated for the simultaneous detection and quantification of nine mycotoxins in seven types of commercial PBMAs; oat, peanut, rice, cashews, soybean, maize and coconut milk (validation parameters are reported in Table 1). The main contaminants found in the samples were aflatoxins and ochratoxin A, but both were within the acceptable range determined by the European Commission regulations. According to the authors, the advantages of this method include its simplicity, speed and lower cost compared to methods using immunoaffinity columns. These benefits make it an efficient and suitable approach for routine analyses (Pinto et al., 2021).

#### 2.2.2 ELISA-based methods to detect mycotoxins in PBMAs

The majority of methods assessing mycotoxin occurrence in PBMAs have employed LC/MS or HPLC coupled with fluorescence. While highly effective in lab settings, these methods are less practical for rapid on-site quality control. Enzyme immunoassays offer a faster alternative and have been successfully used for years to detect aflatoxin M1 in cow milk. Therefore, in addition to instrumental analytical methods, several studies have used immunological methods to detect mycotoxins in PBMAs.

An enzyme immunoassay, a direct method without any need for sample extraction, was recently used to detect mycotoxins in 54 commercial PBMAs samples (Rehagel et al., 2022a). Three out of 54 samples were found to contain mycotoxins, including aflatoxin B1 (AFB1), sterigmatocystin, ochratoxin A, deoxynivalenol and T-2/HT-2 toxin. However, result reliability was negatively impacted by the PBMA matrix interfering with the enzyme immunoassay. To circumvent this issue, a 1:8 dilution of PBMAs was recommended with the possible drawback of compromising the sensitivity of the method and increasing the risk of false-positive or false-negative results. The range of the LODs for this method are reported in Table 1.

To address the issue of high rates of false positives and negatives associated with the traditional Enzyme-Linked Immunosorbent Assay (ELISA) methods, Xu et al. (2021) have recently introduced the indirect competitive Metal-Organic Frameworks Linked Immunosorbent Assay (MOFLISA), a highly sensitive novel methodology for the high-throughput detection of AFB1. This innovative method replaces the natural enzymes with functional metal organic frameworks (MOFs) to catalyse a chromogenic reaction. Compared to conventional ELISA, the developed MOFLISA method exhibited a twenty-fold improvement in LOD values (Table 1). These findings indicated that the recovery rate and accuracy of this detection method surpassed those of conventional ELISA, thereby mitigating the risks associated with false positive and false negative outcomes (Xu et al., 2021).

A sensitive and specific strip test assay based on a competitive immunoassay analysis for the detection of aflatoxins in soy milk has also been developed by Santos et al. (2017). As part of their study a monoclonal antibody named 3B6 was produced exhibiting high specificity to AFB1, B2, G1, G2, and M1 mycotoxins. The developed strip test could detect as little as 0.5 μg/kg of aflatoxin in soy milk. Application of the developed test to soy protein and soy milk from various local markets demonstrated its ability to accurately and qualitatively identify aflatoxin-contaminated samples in less than 10 min. This method offers a promising tool for routine monitoring during the storage, transport, processing and handling of agricultural commodities (Santos et al., 2017).

### 2.3 Pesticide residue identification

Raw plant sources used in PBMA production can be contaminated with harmful chemicals due to standard agricultural and post-harvest practices. Concentration of the raw material during processing often results in these chemicals accumulating in the final commercial product. Therefore, it is essential to monitor chemical contaminant levels in PBMAs to ensure they comply with regulatory limits and tolerances, to protect consumers from the known health risks associated with pesticide exposure. The following section reviews methods for detecting trace levels of pesticides and other toxic elements in PBMAs (Gionfriddo et al., 2020). An overview of the different methods proposed for pesticide determination in PBMAs is provided in Table 2.

**Table 2:** A summary of pesticide determination methods for PBMAs.

Analytical method	Sample preparation technique	LOQ	RSD (%)	Pesticides	References
GC-MS/MS	SPME	1–2.5 μg/kg	<24.5	Trifluralin, Dimethoate, Diazinon, Malathion, Chlorpyrifos, Thiabendazole, Phosalone, λ-Cyhalothrin, β-Cyfluthrin, Esfenvalerate	Gionfriddo et al. (2020)
	SPE	1 μg/kg	NR	Diazinon, Malathion, Chlorpyrifos, Cyfluthrin	Hunter (2009)
	SweEt	2–5 μg/kg	0–30 %	Fipronil, sulfone, piperonyl-butoxide, Pirimiphosmethyl	Giugliano et al. (2023)
GC-ECD	SPE	1 μg/kg	NR	Diazinon, Malathion, Chlorpyrifos, Cyfluthrin	(Hunter, 2009; Lopez et al., 2020)
HILIC-MS/MS	diluted, shaken and centrifuged before extra dilution	10 μg/kg	1.6–26.4	AMPA, Bromide, Chlorate, Ethephon Fosetyl, Glufosinate, Glyphosate, HEPA, MPPA, N-ac-AMPA, N-ac-glufosinate 8, N-a-glyphosate, Perchlorate, Phosphonic acid	Lopez et al. (2020)
UHPLC-MS/MS	QuEChERS	10–25 μg/L	19	Chlorpyrifos	(May et al., 2017)
ECL immunosensor	NR	8.66^a^ pg/mL	NR	Glyphosate	Guerrero-Esteban et al. (2021)
Electrochemical detection using Fe-CuV	NR	5 nM	2.63–2.85	Carbendazim	Yamuna et al. (2021)

NR: not reported.

a LOD.

#### 2.3.1 Chromatographic methods

Chromatographic techniques including gas chromatography (GC) and liquid chromatography (LC) have become the most popular methods for detecting and quantifying pesticides in food matrices due to their sensitivity, separation and identification capacities. Pesticides residues such as fipronil sulfone, piperonyl-butoxide and pirimiphos-methyl have been detected using GC-MS/MS in commercial PBMAs including soy, oat and rice drinks (Giugliano et al., 2023). In another study, GC-MS was paired with solid phase microextraction (SPME) fibre as the sample preparatory method (Gionfriddo et al., 2020). The researchers compared the performance of different coatings for the SPME fibre, a commercial and a novel design; the commercial coating being Polydimethylsiloxane/Divinylbenzene (PDMS/DVB) (RSD = 8.4 %–42.8 %) and the novel one being PDMS/DVB/PDMS (RSD = 24.5 %). The novel methodology exhibited several benefits including the simplification of the sample preparation stage, improved repeatability and suitability for direct-immersion SPME. As a result, it has been proposed as a useful alternative to other extraction approaches executing high-throughput quantitative analysis of pesticides in PBMAs (Gionfriddo et al., 2020).

A particularly challenging group of pesticides in PBMAs are glyphosate-based herbicides which are difficult to analyse using multiresidue methods (methods that target multiple groups of pesticides) due to their high polarity, which hinders extraction and retention in reversed-phase chromatography. To overcome these issues, polar solvents like water and methanol are used for extraction followed by analyses with hydrophilic interaction liquid chromatography (HILIC) which can improve the retention time and analytical peak shape for these types of pesticides. Lopez et al. (2020) developed a HILIC-MS/MS method with minimal sample preprocessing for identifying 14 pesticides and metabolites in oat, rice and soya milk. The results showed the successful determination of 12 out of 14 pesticides in spiked PBMAs samples with a LOD of 10 μg/kg (Lopez et al., 2020).

#### 2.3.2 Alternative methods

Apart from chromatographic-based methods, alternative methods have also been introduced for detecting pesticides in PBMAs, as shown in Table 2. A novel electrochemiluminescence (ECL) immunosensor utilising electro grafted carbon nanodots (CND) successfully detected glyphosate -a widely employed and non-selective herbicide-in soy milk with high sensitivity (Guerrero-Esteban et al., 2021). The ECL immunosensor employed in this study detected glyphosate over a wide linear range from 28.9 to 200 pg/mL, with a sensitivity of 3.38 × 10^−3^ mL/pg and LOD of 8.66 pg/mL. The immunosensor's response proved stable and reproducible, and it was effectively applied to detect glyphosate in tea and soy milk, yielding results consistent with those obtained using an ELISA kit employing the same immunoreagents (Guerrero-Esteban et al., 2021).

Another recent study explored the determination of the highly toxic fungicide carbendazim (CBZ) using iron-doped copper vanadate (Fe-CuV) in soy milk which is a highly sensitive and selective electrochemical method (Yamuna et al., 2021). The material properties of Fe-CuV were comprehensively characterized using X-ray diffraction (XRD), Raman spectrometry, X-ray photoelectron spectroscopy analysis, High-Resolution Transmission Electron Microscopy (HRTEM), and Selected Area Electron Diffraction (SAED) pattern. The effectiveness of the electrode material was further demonstrated through its application in detecting carbendazim in soy milk samples, yielding promising results with a favourable LOD and RSD (Table 2). Consequently, the proposed method may hold potential for utilisation in the development of rapid onsite sensors for the detection and quantification of unwanted and harmful pollutants (Yamuna et al., 2021).

### 2.4 Testing for toxic elements, heterocyclic amines and polycyclic aromatic hydrocarbons in PBMAs

Industrial activities can release heavy metals and metallic compounds into the soil, water and air. These pollutants enter the food chain in different stages, from raw materials to final products posing health risks to consumers. Regular monitoring of these chemicals in foods and beverages is necessary to ensure food safety and to offer consistent nutritional guidance. A few studies have also used the same methodologies to simultaneously detect the presence of micronutrients and heavy metals in food products. Therefore, the following section reviews conventional and novel approaches for detecting both toxic elements and micronutrients in PBMAs.

Table 3 presents a summary of analytical methods and their validation parameters for detecting these elements in PBMAs. For instance, soybean, coconut, and almond milks have been studied for the determination of Na, Mg, K, Ca, P, Fe, Cu, B, Mn, Zn, Al, S, As, Bi, Cd, Co, Cr, Mo, Ni, Pb, Pt, Sb, Se, Sn, Ti, W and Hg elements using microwave–optical emission spectrometry (Karasakal, 2011). Multi-element microwave digestion and inductively coupled plasma mass spectroscopy (ICP-MS) were performed for their determination. Na, Mg, K, Ca, P, S, Mn, Zn, Cu, B, Sb and Sn elements were found in all PBMA samples with K and Mg exhibiting the highest and the lowest mean concentration respectively (Karasakal, 2011).

**Table 3:** A summary of micronutrient and toxic element determination methods for PBMAs.

Analytical method	Toxic and micronutrient elements	LOD	LOQ	RSD%	Ref
**Microwave–OES**	Na, Mg, K, Ca, P, Fe, Cu, B, Mn, Zn, Al, S, As, Bi, Cd, Co, Cr, Mo, Ni, Pb, Pt, Sb, Se, Sn, Ti, W, Hg	0.17–17 μg/L	5–25 μg/L	<2	Karasakal (2011)
**ICP OES**	Al, As, Cd, Co, Cr, Cu, Fe, Li, Mn, Ni, Pb, Sb, Se, Sn, Sr, Zn	0.3–12 μg/L	1.1–38.2 μg/L	<10	Milani et al. (2023)
	Ag, Al, Ba, Ca, Cd, Co, Cr, Cu, Fe, In, Mg, Mn, Ni, Pb, Zn	0.03–1.08 mg/kg	0.10–3.23 mg/kg	0.4–10.8	Manousi and Zachariadis (2021)
**ICP-MS & NAA**	As, Cd, Co, Cr, Cu, Fe, Mn, Mo, Ni, Pb, Se, V, Zn	R	NR	NR	Chajduk et al. (2018)
**ICP-MS**	Tl, Sb, Th, Pb, Cd, Co, As, U, Se, Mo, Li, Cr, Ni, Ba, Cu, Al, B, Fe, Sr, Zn, Mn, Mg, Na, Ca, K	NR	NR	NR	Godebo et al. (2023)

∗NR: Not reported.

ICP-MS combined with neutron activation analysis (NAA) was also employed to analyse the elemental contents of commercial PBMAs in commercial brands available in the Polish market (Chajduk et al., 2018). Specifically, the concentration of both toxic and essential elements was measured in soy and goat-based milk infant formulas. NAA using radiochemical mode as a complementary method was applied to measure arsenic and it was found that PBMAs were either free of toxic elements or contained very low amounts within the limits considered safe for infants (Safety Levels of Toxic Elements in processed cereal-based foods for infants 20–100 μg/L). Importantly, the trace elements detected in both plant and animal-based milks were in agreement with the values stated on the products’ labels (Chajduk et al., 2018). A study in the USA (Godebo et al., 2023) analysed 25 elements present in PBMAs and other plant-based drinks using ICP-MS. Most elements were detected at levels below the drinking water standards, however seven out of the 25 elements surpassed these standards with aluminium and zinc exceeding the secondary, non-enforceable drinking water standards (World Health Organization WHO, 2011; (Godebo et al., 2023).

Another study detailed the development and validation of an Inductively Coupled Plasma Optical Emission Spectroscopy (ICP-OES) technique for quantifying essential nutrients and toxic metals in PBMAs (Manousi and Zachariadis, 2021). The analysis covered a range of trace elements for various commercial PBMAs available in Greece, including almond, walnut and peanut PBMAs. The validation parameters for this method are presented in Table 3. The findings highlighted the reliability and efficacy of the developed analytical approach for assessing the metal content in various PBMAs (Manousi and Zachariadis, 2021). ICP-OES was also used to detect trace elements in soy-based milk following an i*n vitro* digestion and cell culture bio-accesibility assay. Although bio-accessibility of seven elements; Al, Cu, Fe, Mn, Sr, Se and Zn was between 40 and 80 % of their total content, their findings indicated that the daily consumption of one glass of soy-based beverage could account for 3.5 % of the Provisional Tolerable Weekly Intake (PTWI) for aluminium in children and 0.9 % in adults (Milani et al., 2023).

Another safety concern for PBMAs is the presence of heterocyclic aromatic amines (HAs) and polycyclic aromatic hydrocarbons (PAHs). HAs are readily generated when protein-rich foods undergo thermal processing while PAHs have been identified in the leaves and stems of plants cultivated in PAH-contaminated regions, which is their entry route to PBMAs. HAs and PAHs exhibit carcinogenic, highly mutagenic properties and pose potential risks to human health (Alaejos and Afonso, 2011) making their detection in food products a crucial endeavour (DiScenza et al., 2018).

HAs were analysed using UPLC-MS/MS with QuEChERS employed for sample extraction in soy milk and tofu products prepared through various cooking methods with a reported LOD of 0.003–0.100 ng/g and LOQ of 0.010–0.350 ng/g. The levels of HAs in the samples demonstrated an escalation with both cooking temperature and cooking duration. Notably, 9H-pyrido [3,4-b] indole (Norharman) and 1-methyl-9H-pyrido [3,4-b] indole (Harman) were identified as the primary contributors to the HAs content across all samples (Chiang et al., 2022). In another study, ten PAHs and PAH metabolites in various types of PBMAs (almond, cashew and soy) and bovine milk were investigated using cyclodextrin-promoted energy transfer, which relies on fluorescence energy transfer from the PAH to a high quantum yield fluorophore, coupled with subsequent array-based statistical analyses of the fluorescence emission signals (DiScenza et al., 2018). Notably, this system demonstrated effective sensitivity (detecting low micromolar concentrations, LOD = 0.09–45.11 μM), selectivity (with 100 % differentiation even among structurally similar analytes) and broad applicability (suitable for unmodified lipophilic PAHs, highly polar oxidized PAH metabolites and different types of PBMAs). These encouraging findings hold promise for the development of solid-state devices for the detection of PAHs and their metabolites in complex, commercially available food products (DiScenza et al., 2018).

## 3 Detection of allergens in PBMAs

Food allergies, triggered by allergenic compounds present in foods and beverages, represent hypersensitivity immune responses in humans with potentially severe consequences. The increasing popularity of plant-based and lactose-free diets has led to a greater consumption of PBMAs, raising concerns about cross-contamination with allergenic proteins during manufacturing (Xiao et al., 2023). PBMAs allergens include legumes such as soybean or pea, tree nut such as hazelnut proteins and cashews, gluten sources and some additives such as xanthan gum or guar gum. Around 16 soybean allergens have been previously identified and characterized in soy milk and other commercial foods; among these, 7 S globulin (Gly m 5), 11 S globulin (Gly m 6 or Glycinin), Gly m Bd 28 K, and Gly m Bd 30 K stand out as the primary soybean allergens (Krishnan et al., 2009; Ladics et al., 2014; Murakami et al., 2018). Soybean Gly m Bd 28 K, derived from Gly m 5, exhibits strong IgE-binding capacity with antibodies from individuals sensitive to soybeans. Additionally, profilin (Gly m 3) and SAM22 (Gly m 4) are classified as pathogenesis-related proteins with immunological characteristics similar to fruit pollen allergens like Bet v1 and Bet v2. Gly m 4 are susceptible to heat, acids and proteases treatments potentially remaining undetected in processed foods (Ueberham et al., 2019). In contrast, the minor storage protein 2 S albumin (Gly m 8) demonstrates higher thermal stability and resistance to protease digestion, playing a pivotal role in diagnosing severe allergies in children (Zhu et al., 2022). In this section conventional and novel methods to detect allergen in PBMAs have been reviewed.

DNA-based assays, HPLC and immunoblotting assays are commonly and routinely employed in research and testing laboratories for the detection of soybean allergens (Houston et al., 2011; Scharf et al., 2013; Wang et al., 2017; Yuan et al., 2018). DNA-based assays such as PCR and loop-mediated isothermal amplification (LAMP) target specific regions of soybean DNA. DNA is highly resilient and minimally affected by thermal processing, thus successfully employed in detecting soybean allergens in processed food products. Nevertheless, the detectability of soybean DNA has been reported to decrease during food processing and cross-reactivity with several foods including barley and buckwheat has been observed (Bauer et al., 2011; Costa et al., 2017; Galan et al., 2011). The ELISA method relies on the interaction between specific antigens and their antibodies, commonly used for detecting food allergens due to its high throughput, sensitivity and lower cost (Asensio et al., 2008). A specific sandwich ELISA method with two detection and capture antibodies was used to detect multiple soybean allergens in soy milk (and other commercial foods with and without soybean labels on the package). The specification and quantification range of this method were higher than the common ELISAs with antibodies based on a single allergen. The accuracy and precision of this method was also tested with samples spiked with different levels of soybean content (Table 4) and some food samples without soybean specified in the label were found to contain soybean residues (Zhu et al., 2022). HPLC-MS/MSoffers the benefits of high throughput and accuracy; however, it requires expensive equipment and peptides of high purity. A different study combined LC–MS/MS and a stable-isotope dimethyl labelling method in soy milk to determine Gly m 6 throughout various food processing methods. The Gly m 6 content was compared in raw, after natto fermentation, pasteurization and sterilization of soy milk, and this allergen was shown to decrease by 42 %, 31 % and 35 % respectively following these treatments. However, a significant portion (19 %) of Gly m 6 from raw soy milk was still preserved in the soy milk film. This research expanded the applicability of dimethyl labelling to soy-based food samples and scrutinised the proteolysis of Gly m 6 during natto fermentation and its susceptibility to thermal instability (Hsiao et al., 2022). A particular challenge in allergen detection in PBMAs is the modification of allergen residues during manufacturing processes (both UHT and UHPH treatments) inducing protein aggregate formation, which reduces the effectiveness for antibody-based detection methods (Kato et al., 1983; Poliseli-Scopel et al., 2012; Sorgentini et al., 1995; H. Zhang et al., 2004). To overcome this challenge, a panel of polyclonal antibodies targeting modified proteins in almond, cashew, coconut, hazelnut and soy milks was developed by Masiri et al. (2016). These antibodies were incorporated into rapid lateral flow immunoassay tests, designed in both sandwich and competitive formats. Tests exhibited robust detection capabilities across various PBMA products, with high sensitivity, rapid results (under 25 min) and minimal cross-reactivity with extracts from common commodities (Table 4) (Masiri et al., 2016).

**Table 4:** A summary of allergen determination methods for PBMAs.

Analytical method	Allergens	LOD	RSD%	Ref
Common ELISAs	β-conglycinin, Gly m Bd 28 K, Gly m Bd 30 K, Gly m 6	0.02–2.5 ppm	NR^a^	Zhu et al. (2022)
Specific sandwich ELISAs	β-conglycinin, Gly m Bd 28 K, Gly m Bd 30 K, Gly m 6	0.08–0.25 ppm	<16.67	Zhu et al. (2022)
Lateral flow immunoassay tests	Modified β-conglycinin, 2 S albumin, Gly m 6	1ppm	NR	Masiri et al. (2016)
LC-MS/MS	Gly m 6	5.4 ng/mL	NR	Hsiao et al. (2022)
SMARTPHONE-iSPR biosensor	Total hazelnut protein	0.04–0.53 μg/mL	4.21–11.08	Xiao et al. (2023)
BENCHTOP SPR biosensor	Total hazelnut protein	0.02–0.04 μg/mL	0.18–0.9	Xiao et al. (2023)
Aptamer sensor	β-Conglycinin	2.58 nm	NR	Ma et al. (2022)

a NR: Not reported.

Traditional allergen screening methods, typically performed in laboratories, can also be complemented by portable biosensors for on-site screening in production facilities to enhance quality control and food safety. In a study by Xiao et al. (2023) a portable smartphone imaging surface plasmon resonance (iSPR) biosensor featuring a 3D-printed microfluidic surface plasmon resonance (SPR) chip was introduced for detecting total hazelnut protein in commercial PBMAs. The iSPR and conventional benchtop SPR systems were compared, revealing comparable characteristic sensorgrams (real-time interaction data) but the benchtop system had a much lower %RSD compared to the smartphone iSRP system when detecting trace levels of total hazelnut protein in spiked PBMAs (Table 4). Nevertheless, the portability and miniaturized nature of the smartphone iSPR biosensor platform holds promise for on-site food allergen detection by food producers in the future (Xiao et al., 2023).

β-Conglycinin is another food allergen which poses challenges for the existing detection methods. Ma et al. (2022) developed an accurate and sensitive aptamer sensor for the rapid detection of β-conglycinin, leveraging the specific binding properties of the β subunit of β-conglycinin and its aptamer. The sensor exhibited excellent performance in terms of sensitivity and detection efficiency, with a detection range for the β subunit of 7–58 nM and a detection limit of 2.58 nM. Validation of the sensor was achieved by successfully detecting additional β subunit in soy milk, demonstrating its adaptability to a complex matrix. These results underscore the potential of aptamer sensors as novel tools for ensuring the quality control of soybean meal products (Ma et al., 2022).

## 4 Discovery of ambivalent compounds in PBMAs

The term “ambivalent compounds” in the context of this study refers to substances in food products that can exhibit both beneficial and detrimental health effects. An example of ambivalent compounds are phytoestrogens, a group of natural non-steroidal plant compounds which include various subgroups such as flavonoids, lignans and coumestans. Phytoestrogens have been reported to possess antioxidant properties, potential anti-carcinogenic effects, protection against heart disease by lowering cholesterol and relief from menopausal symptoms. However, due to their estrogenic activity, they are also classified as endocrine-disrupting chemicals (Gopaul et al., 2012; Le Fur et al., 2000; Rietjens et al., 2013).

Several studies have investigated the detection of phytoestrogens in PBMAs using UHPLC, GC–MS/MS and sensor-based methods. For instance, a UHPLC method coupled with UV detection was developed for the simultaneous determination of 12 isoflavones in less than 8 min in eight commercial soy milks with sensitivity (LOD) and precision (RSD) parameters reported in Table 5. Commercial soy milk samples had a total isoflavone content between 1805.22 and 3195.59 mg/kg dw, with genistin and daidzin being the major isoflavones across all samples (Toro-Funes et al., 2012). In another study, a fast UHPLC-ESI-MS/MS method combined with one-step (40 min) salting-out assisted liquid–liquid extraction (SALLE) was developed and validated for high throughput analysis of 12 isoflavones in different soy milks. A great variation (3.7-fold) was observed in the contents of isoflavones in 22 commercial soy milks with concentrations ranging 74.3–273.6 mg/L (mean, 162.7) (Park and Jung, 2017).

**Table 5:** A summary of ambivalent compounds detection methods for PBMAs.

Analytic method	Ambivalent compounds	LOD	RSD%	Ref
UHPLC & UV	daidzin, glycitin, genistin, daidzein glycitein, genistein, malonyl daidzin, malonyl glycitin, malonyl genistin, acetyl daidzin, acetyl glycitin, acetyl genistin,	0.05 mg/L	<5	Toro-Funes et al. (2012)
UHPLC-ESI-MS/MS (SALLE)	daidzin, glycitin, genistin, daidzein glycitein, genistein, malonyl daidzin, malonyl glycitin, malonyl genistin, acetyl daidzin, acetyl glycitin, acetyl genistin,	1–30 pg	<5.3–5.6	Park and Jung (2017)
GC–MS/MS	formononetin, daidzein, coumestrol, genistein, and biochanin A	0.1–17.7 μg/L	4.7–6.1	Benedetti et al. (2018)
Fluorescent sensor	genistein	35.7 nM	0.13–5.03	Zhang et al. (2023)

Several methods employing GC–MS have also been proposed for analyzing phytoestrogens in food (Ferrer et al., 2009; Liggins et al., 2002). These methods require a derivatisation step since phytoestrogens are slightly polar compounds with low volatility, making them unsuitable for direct analysis using GC–MS. Despite this, GC–MS continues to be a widely applied technique, as it is more cost-effective compared to LC-MS and could offer potential advantages in terms of sensitivity and specificity. The derivatisation process is a critical aspect of this methodology as it can be challenging to optimise conditions to ensure speed, reproducibility and efficiency (Liscio et al., 2009; Magi et al., 2010). Benedetti et al. assessed three different silylation reagents with the aim to optimise the derivatisation process of five phytoestrogens in soy milk prior to GC–MS/MS analysis. The validation parameters of this method are reported in Table 5 showing excellent sensitivity compared to LC-MS based methods (Benedetti et al., 2018).

The rapid detection of genistein in soy milk powder, and soybean milk has also been investigated using a straightforward one-pot reverse microemulsion method. This method has been employed to synthesise a sensor based on N-doped carbon dots conjugated with molecularly imprinted polymers for genistein analysis. N-doped carbon dots served as the fluorescent component, genistein as the template molecule, and molecularly imprinted polymers as the selective sorbent in this fluorescence sensor. To validate this method, HPLC was employed, revealing no significant differences between the fluorescence analysis and HPLC results. The results also indicated that the developed sensor was an effective fluorescent tool for detecting genistein in complex food samples (Zhang et al., 2023).

## 5 Testing for adulterants in PBMAs

Adulterants in PBMAs can refer to all accidental or intentional addition of materials and ingredients not included in the product label. Accordingly, all the types of contaminants mentioned in the previous sections can also be included within the “adulterant detection” category since the source and safety of the adulterant is questionable. In the context of this review, we have focused on detecting PBMA adulteration with other plant species, as reported in previous research work. This practice can result in nutritional losses and potential health risks for the consumers. In addition, adulteration incidences can result in economic losses and harm the commercial reputation of producers and trade labels. Introducing accurate and efficient techniques to detect adulterants in PBMAs is of critical importance. Existing studies focusing on detecting potential adulterants in different PBMAs types have relied on both DNA-based methods and chemical analytical methods.

### 5.1 DNA-based methods for detection of PBMA adulterants

Polymerase chain reaction (PCR) is a common technique used in food authentication, which amplifies a specific region of DNA to confirm the presence or absence of the target DNA fragment belonging to the investigated adulterant. Quantitative (q)PCR, digital PCR such as droplet digital (dd)PCR and DNA barcoding and metagenomics are advanced methodologies based on conventional PCR. These techniques are designed to enhance the precision and accuracy of tracking and quantifying amplification targets. qPCR allows for real-time quantification of DNA during the PCR process, enabling researchers to measure the DNA quantity in a sample at each cycle. DdPCR takes this a step further by partitioning the PCR reaction into thousands of individual droplets, providing absolute quantification of DNA molecules without the need for standard curves. DNA barcoding, on the other hand, utilises short, standardised DNA sequences to assign taxonomy, making it a powerful tool for biodiversity studies and species identification. Metagenomics enables the comprehensive analysis of microbial communities without the need for culturing individual organisms. Together, these methods represent significant improvements over conventional PCR, offering greater sensitivity, specificity and quantitative capabilities in food authenticity applications (Galimberti et al., 2013; Lanubile et al., 2024).

Several studies have used these DNA-based methods to detect adulterants in PBMAs. For instance, ddPCR has been used to quantify the presence of adulterants in walnut milk using the lectin gene and the walnut Jugr2 gene as targets (Yang et al., 2017). The method utilised the ratio between the numbers of target gene copies per unit mass of walnut and soybean to reveal the relationship between gene copy number and the mass of plant materials. The LOD and relative error for added soybean-derived ingredients in walnut beverage were 0.5 % and 5.6 %, respectively. Application of this method on eleven commercial walnut milks, revealed that four walnut milks were adulterated with more than 10 % soybean and seven samples contained as low as 0.2 % soybean (Yang et al., 2017).

DNA barcoding methods are increasingly used for quality assurance of food samples with several chloroplastic and non-chloroplastic DNA regions being proposed as DNA barcodes for discriminating plant speciation in food samples such as PBMAs. *RbcL*, *trnL, matK*, *rpoB*, *psbA-trnH*, and *psbK-I* are chloroplastic DNA regions which can be used as DNA barcodes for differentiating plant taxonomies (Matiz-Ceron et al., 2022). Non-chloroplastic-nuclear-DNA barcodes, such as an internal transcribed spacer (ITS), have also been tested in plant species (Ahmed et al., 2022; Antil et al., 2023). For instance, Ding et al. (2020), used *psbA-trnH* as a marker to differentiate plant taxa as this marker gene has a faster evolution rate and higher efficiency in identifying plant species. Matiz-Ceron et al. (2022), reported that *trnL* and *matK* can be more effective candidate markers to differentiate with resolution up to genus level. *MatK* along with *rbcl* have been reported as effective barcodes in various other studies focusing on species determination (Ding et al., 2020; Feng et al., 2018; Bolson et al., 2015). The ITS region, especially ITS2, is considered a highly effective DNA metabarcoding marker for plants due to its strong discriminatory power from high interspecific variation and fewer challenges with amplification and sequencing compared to plastid barcodes (Omelchenko et al., 2019). The evidence so far suggests that a combination of markers is a better solution for identifying all the plant species within a food sample as there may be no single barcode able to achieve the same goal (Ahmed et al., 2022; Antil et al., 2023).

Regarding PBMAs, *rbcL-4* and *matK-4* have been used previously to detect adulterant ingredients in walnut milk (Han et al., 2018). It was found that these two primers could amplify related genes in walnut, peanut, sesame, soybean and hazelnut. To test the specificity of this method, different proportions of genomic DNA from soybean and peanut were added to the walnut milk, and it was found that primer *rbcL-4* is capable of detecting peanut genome DNA at a 10 % concentration, while primer *matK-4* can identify soybean genome DNA at the same level. Based on extraction rate calculations*, rbcL-4* can detect the presence of 8.88 % peanut raw material, and *matK-4* can detect 2.30 % soybean raw material. This study demonstrated that these two universal primers can be used together to provide reliable results on PBMA authenticity (Han et al., 2018).

DNA barcoding alongside high-resolution melting (HRM) analysis (quantitative analysis of melt curves of amplicon products) have also been used for the detection of adulterants in PBMAs without the need for amplicon sequencing. These two methods can be high-throughput, cost-effective, rapid (approximately 3 h) and reliable (Caldwell, 2017; Costa et al., 2016; Osathanunkul, 2018; Pereira et al., 2018; Sun et al., 2016). HRM analysis targeting the *psbA*-*trnH* barcode can produce standard melting profiles of different tree nut species, and, in combination with DNA barcoding, it can be used for both qualitative and quantitative detection of nut adulterants in walnut milk. Since melting curve peaks and HRM analysis were different for each nut species, this method showed promise for adulterant detection in other high-value food products such as olive oil, wine and herbal medicines (Degtjareva et al., 2012; Ding et al., 2020; Pang et al., 2012).

### 5.2 Analytical methods for the detection of PBMA adulterants

Apart from DNA-based methods, analytical methods such as chromatographic or mass spectrometry methods, can also be used to detect adulterants in PBMAs. Hyphenated techniques such as HPLC-MS and GC-MS, are valuable analytical tools for detecting food adulteration. Chromatography excels in separating and identifying the individual components of a food sample, while mass spectrometry offers details about the molecular structure and concentration of these components. Together, these techniques are instrumental in identifying and quantifying unwanted substances in food products, playing a crucial role in ensuring their safety, quality and compliance with regulatory standards.

HPLC-DAD (diode array detector) has also been successfully employed for detecting adulterants in five commercial PBMAs types (Zhang et al., 2013). Specifically, walnut milk adulteration with soybean was detected by separating and identifying soybean isoflavones using HPLC-DAD with LODs 0.03–0.1 μg/mL and %RSD of 4.62. Another study was the first to employ desorption electrospray ionization mass spectrometry (DESI-MS) to analyse, detect and classify biomarkers of lipids in milk from five different animal and plant sources (Hong et al., 2022). Classifications of bovine milk, goat milk, camel milk, soya milk and oat milk were further achieved with a 100 % cross-validation rate using linear discriminant analysis (LDA). This method exhibited high classification accuracy while eschewing the need for complex sample preparation (Hong et al., 2022).

England and coworkers (2020) used lipid fingerprinting with routine matrix-assisted laser desorption/ionization (MALDI)-Time of flight (TOF)-MS and principal component analysis (PCA) classification to characterize PBMAs. Phosphatidyl choline was found to be the most prominent peak for soy and bovine milk, while triacylglycerols were the dominant lipids for coconut milk (England et al., 2020). Hyperspectral imaging and convolutional neural networks (CNN) have also been used to distinguish animal-based milk adulteration (bovine and goat) in soy and coconut milk with a high degree of accuracy and minimal computational time. These findings demonstrated that hyperspectral imaging combined with CNNs can be effective for classifying different types of milk either by the type, organism or processing procedure (fresh and UHT) (Asmara and Saputro, 2022).

## 6 Discussion

In recent years, consumption of PBMAs has increased dramatically, while control methods to guarantee their safety have yet to be developed accordingly. It is therefore imperative to identify prospective contaminants, allergens and adulterants in PBMAs and develop effective detection methods. This review provided a comprehensive overview of all types of potential adulterants, contaminants, allergens and ambivalent compounds in PBMAs. It addressed both conventional and emerging detection methods by presenting their advantages and limitations.

Adulterants are sometimes introduced into PBMAs to artificially enhance certain parameters, misleadingly boosting the perceived quality of the product. However, there has been limited research focused on this issue. Peanut and soybeans have been already identified as potential adulterants in PBMAs, but further investigation is needed to uncover additional potential adulterants, such as foreign proteins, sugars, adulteration with different PBMA sources, chemical compounds, added water, GMOs and the proportion of various ingredients. Expanding the scope of these studies will help ensure the safety and integrity of PBMAs by identifying any additional adulterants that could pose health risks to consumers or compromise product quality.

In comparison, numerous studies have been conducted to detect a wide range of adulterants in animal milks. These adulterants include diluents such as water and milk whey, non-protein nitrogen compounds like melamine and urea, and substances used to extend product shelf life, including hydrogen peroxide, formaldehyde and salicylic acid. Additionally, adulterants used to alter fat content, such as animal fat, vegetable oil and surfactants have also been identified (Ceniti et al., 2023; Choudhary and Sharma, 2024; Ionescu et al., 2023; Poonia et al., 2017). Previous studies have also focused on milk adulteration (e.g. bovine milk) with milk from different animal species and have highlighted the variety of adulteration practices in these products (Mafra et al., 2022). In the case of PBMAs, there is a noticeable lack of such comprehensive studies and corresponding detection methods. Moreover, regulatory agencies worldwide have set maximum residue limits (MRLs) and tolerable daily intakes for certain contaminants in animal milk. However, despite the health risks posed by contaminant residues in PBMAs, MRLs have not yet been established for all potential contaminants, adulterants and allergens.

DNA-based technologies, with enhanced reliability, sensitivity and high-throughput capabilities offer superior solutions for assessing the authenticity of plant-derived foods. The detection of adulterants in PBMAs primarily relies on DNA-based techniques, including conventional PCR, qRT-PCR, ddPCR, DNA barcoding (using various markers) and DNA barcoding combined with HRM analysis. Since the DNA is specific to each individual organism and unaffected by environmental factors the DNA-based methods provide greater accuracy for species attribution and variety identification compared to other types of analyses (Catalano et al., 2016). Additionally, improvements have been done for commercial DNA extraction kits improving their efficacy for DNA extraction from both fresh and processed food samples with complex matrices.

Despite advancements in technology and instrumentation, DNA-based approaches still face challenges and limitations, particularly due to the critical importance of DNA extraction and the removal of metabolic inhibitors for downstream analyses. For example, the production of PBMAs involves various treatments—such as grinding, heating, enzyme treatment and UHT processing—which could affect DNA content, integrity and quality. Also, DNA extraction might be impaired by the presence of various additives, supplements and secondary metabolites found in food extracts. Co-extraction of PCR inhibitors, such as polysaccharides and polyphenolics, can interfere with the PCR process by binding to DNA or blocking the magnesium cofactor of the PCR buffer and disrupting amplification results (Lanubile et al., 2024; Lo and Shaw, 2018). Therefore, optimal sample preparation and DNA extraction protocol optimisation are critical steps for the success of DNA-based methods. In this context, nanoparticles and microfluidic tools have been explored by several researchers to enhance the accuracy and efficiency of DNA isolation and purification in various food sources, including grapevine products and olive oil (Carvalho et al., 2018; Teixeira et al., 2023). The significance of extraction protocols in complex matrices, such as olive oil, was also examined by (Scollo et al., 2016), however, similar studies have yet to be initiated for PBMAs.

DNA barcoding addresses some of the challenges associated with focusing on one or more specific genomic regions rather than investigating available and complete genome sequences for different targets. DNA barcoding often utilises the mitochondrial genome, which is less likely to be damaged during industrial food processing. However, this method is not without its limitations. A major drawback is that it is impossible to generate universal primers or genes which can be amplified in all organisms to provide sufficient sequence variations for accurate species identification (Antil et al., 2023). A further challenge involves selecting appropriate DNA targets with suitable primers and ensuring their availability in PBMA samples. An alternative approach could be to use techniques which focus on the chloroplast genome, which has a higher copy number in plant cells, instead of relying on low copy number genes of the nuclear DNA (Wu et al., 2023). However, optimizing and establishing a universal marker remains difficult, therefore some studies recommend using a combination of DNA markers to detect adulterants in processed products. A significant advancement in this area is the ever-expanding genomic databases containing fully sequenced genomes of plant and animal species which can help generate more appropriate markers (Fanelli et al., 2021).

Recently developed technologies have greatly enhanced the effectiveness of DNA-based methods for verifying food authenticity, and these advancements are also applicable to PBMAs. Leveraging recently developed techniques such as DNA metabarcoding and CRISPR/Cas can enhance the efficacy of DNA methods to effectively detect these adulterants in PBMAs. DNA metabarcoding, which combines DNA barcoding with next generation sequencing (NGS) data generation using universal primers, allows the identification of multiple species from complex samples. The term ‘meta' in metabarcoding indicates the compilation of barcode sequences from various species (Ji et al., 2013; Staats et al., 2016). While DNA barcoding identifies individual species from single specimens, metabarcoding provides a broader view of biodiversity and community composition by identifying multiple species from a complex matrix. This novel technology is advantageous for ensuring PBMAs safety as it can simultaneously uncover multiple species in PBMAs using universal primers. It has also attracted considerable attention for food authentication purposes to address issues such as incorrect labelling, substitutions and contamination issues. DNA metabarcoding has been particularly effective in tracing processed food products, especially used for seafood and meat samples. For instance, it has uncovered widespread mislabelling and substitution of fish and seafood products in several restaurants and marketplaces across various countries (Mottola et al., 2022; Noh et al., 2021; Piredda et al., 2022).

Apart from DNA-based methods, a few other analytical methods and classification techniques have been proposed to detect adulterants in PBMAs. These methods mainly focus on distinguishing between animal and plant-based products, such as separating bovine and goat milk from PBMAs. However, there remains a gap in classifying the various types of PBMAs based on their plant sources.

Regarding contaminants, published research has predominantly focused on mycotoxins and chemical contamination in PBMAs, with less attention given to bacterial, viral, fungal, antimicrobial and processing contaminations. The most widespread techniques for detecting PBMAs contaminants include instrumental and immunoassay methods, but these conventional approaches have certain limitations. As a result, in recent years there has been a growing focus on developing rapid, reliable, sensitive, and effective detection techniques for identifying contaminants in complex PMBA matrices.

In the case of microbial contamination of PBMAs, culture-based methods and PCR are routinely used for their detection. There is a notable lack of advanced methodologies for detecting this group of contaminants in PBMAs, such as advanced nucleic acid and sequencing approaches, array-based methods and biosensor technologies.

For mycotoxins detection in PBMAs, analytical methods such as HPLC and LC-MS are the most frequently applied. The distinct physicochemical properties of PBMAs can pose challenges in their extraction efficiency due to the great variation of the analyte structure. To address these challenges, studies have mostly focused on the development of reliable and rapid sample preparation methods for HPLC and LC-MS-based analyses, but novel approaches are also optimised for the simultaneous detection of multiple analytes, which helps reduce costs and minimize operational interventions. Consequently, through a comparison of various detection methods for mycotoxins, LC-MS/MS combined with QuEChERS for sample preparation can be recommended as the most sensitive and precise technique (Arroyo-Manzanares et al., 2019; Pavlenko et al., 2024; Pinto et al., 2021; Rehagel et al., 2022b).

Despite their prevalence, chromatography-based methods may not be practical for rapid analysis of raw materials due to their high cost and the requirement of skilled personnel. To tackle this issue, rapid detection methods using smartphone-based, microfluidic chip-based, biosensors, and paper-based devices have been developed, which integrate electrochemical and optical biosensing platforms as well as electronic noses for the quick detection and measurement of contaminants. These emerging methods are not only fast and portable but also align with the principles of green chemistry, as they often require minimal reagents, produce less waste, and consume less energy compared to traditional laboratory techniques. As such, they are considered more environmentally friendly and sustainable. While these techniques still need to be optimised and assessed for PBMAs analyses, they represent a promising and greener solution for the food industry. Indeed, simplifying protocols and enabling on-site analysis are critical steps in decentralising food testing methods to ensure more robust, eco-conscious, and rapid analyses for the authenticity of PBMAs.

## 7 Conclusions

The rapid expansion of commercially available PBMAs has outpaced the development of effective safety and authentication measures, emphasizing the urgent need for reliable contaminant, allergen and adulterant detection methods. This review provides a comprehensive overview of potential contaminants, allergens, and adulterants in PBMAs, along with the advantages and limitations of various detection techniques. While substantial progress has been made in identifying chemical and biological contaminants, significant gaps remain, particularly in addressing cross-contamination and processing-related issues, such as residues from cleaning agents and packaging materials. Additionally, while extensive research has focused on adulterant detection in animal milk, comparable studies for PBMAs are still limited.

Although emerging detection technologies hold promise, many are not yet fully optimised for routine use in commercial settings. Conventional techniques like chromatography and spectrometry remain the gold standard for detecting contaminants such as mycotoxins and chemical adulterants, however their high cost, complexity, and time-consuming nature limit their practicality for rapid or on-site testing. In contrast, alternative rapid detection methods, including biosensors, smartphone-integrated assays, and microfluidic devices, show great potential for enabling on-site analysis and real-time monitoring of PBMAs.

Two primers were used to detect adulterants in walnut milk by targeting genes from various nuts and legumes, including peanut and soybean. While the method provides reliable results for PBMA authentication, future work should focus on developing more sensitive approaches capable of detecting adulterants at concentrations as low as 1 %.

As the food industry shifts toward sustainability, green analytical methods can become promising solutions for the safety and quality assurance of PBMAs. Eco-friendly, sustainable detection techniques minimize hazardous chemicals, energy consumption, and waste while maintaining sensitivity and accuracy. Methods like solvent-free extraction, water-based chromatography, and energy-efficient miniaturized systems significantly reduce toxic waste and energy use. Biosensors, paper-based assays, and nanotechnology-based sensors provide rapid, cost-effective alternatives. Additionally, AI-driven spectroscopy and sustainable sample preparation techniques, such as microwave-assisted and enzyme-based extraction, enhance detection efficiency while minimizing environmental impact.

To ensure the authenticity, safety, and quality of PBMAs, future efforts should focus on integrating multiple detection approaches, refining greener methods, and addressing current technological limitations. Bridging these gaps will be crucial for meeting regulatory standards, fulfilling commercial and sustainability demands, and ultimately boosting consumer confidence in PBMAs. Standardizing detection protocols and developing scalable technologies will be crucial in ensuring the safety and authenticity of PBMAs.

## CRediT authorship contribution statement

**Zahra Karimi:** Conceptualization, Methodology, Investigation, Visualization, Writing – original draft. **Katrina Campbell:** Conceptualization, Methodology, Writing – review & editing, Supervision. **Zoltan Kevei:** Conceptualization, Methodology, Writing – review & editing, Supervision. **Andrea Patriarca:** Writing – review & editing. **Anastasios Koidis:** Writing – review & editing. **Maria Anastasiadi:** Conceptualization, Methodology, Writing – review & editing, Supervision, Funding acquisition.

## Funding

This research was funded by 10.13039/100014013UKRI 10.13039/501100000268BBSRC FoodBioSystems Doctoral Training Partnership (DTP), grant number BB/T008776/1.

## Declaration of competing interest

The authors declare that they have no known competing financial interests or personal relationships that could have appeared to influence the work reported in this paper.

## Data Availability

No new data were created or analysed during this study. Data sharing is not applicable to this article.

## List of Abbreviations:

AFB1: Aflatoxin B1
CFU: colony-forming units
ddPCR: droplet digital PCR
DESI-MS: desorption electrospray ionization mass spectrometry
DON: deoxynivalenol
DVB: divinylbenzene
ECL: electrochemiluminescence
ELISA: enzyme-linked immunosorbent assays
FLD: fluorescent detection
GMOs: genetically modified organisms
HAs: Heterocyclic amines
HPLC: high-performance liquid chromatography
HRTEM: high-resolution transmission electron microscopy
ICP: inductively coupled plasma
ICP-MS: inductively coupled plasma mass spectrometry
iSPR: imaging surface plasmon resonance
LC–MS/MS: liquid chromatography–tandem mass spectrometry
LDA: Linear discriminant analysis
LOD: Limits of Detection
LOQ: Limits of Quantification
MALDI-TOF: matrix assisted laser desorption ionization
matK: maturase K
MOFs: metal-organic frameworks
MOFLISA: MOF-linked immunosorbent assay
MS: mass spectrometry
NAA: neutron activation analysis
NGS: next-generation sequencing
OES: optical emission spectrometry
OTA: ochratoxin A
PAHs: polycyclic aromatic hydrocarbons
PBMAs: Plant-based milk alternatives
PCA: Principal component analysis
PCR: polymerase chain reaction
PDMS: polydimethylsiloxane
psbA-trnH: rpoB, photosystem II protein D1—Transfer RNA H
psbK-I: Photosystem II K protein—I spacer
qPCR: quantitative PCR
QuEChERS: a solid phase extraction method and the name is a portmanteau word formed from “quick, easy, cheap, effective, rugged, and safe"
rbcl: Ribulose-1,5-bisphosphate carboxylase/oxygenase large subunit or RuBisCO large subunit
RSD: relative standard deviation
SAED pattern: Selected area electron diffraction
SALLE: salting-out assisted liquid-liquid extraction
SPE-HPLC: Solid phase extraction-high performance liquid chromatography
SPME: solid phase microextraction
STC: sterigmatocystin
SweEt: Swedish Ethyl Acetate method
trnL: RNA T—L spacer
UHPH: Ultrahigh pressure homogenization
UHPLC-MS/MS: ultra-high performance liquid chromatography tandem mass spectrometry
UHT: ultra-high temperature
XPS analysis: X-ray photoelectron spectroscopy
XRD: X-ray powder diffraction
